# A Scoping Review of Active Service User Involvement in Undergraduate Allied Health Professions Education

**DOI:** 10.1111/hex.70575

**Published:** 2026-02-04

**Authors:** Chloe Shand, Jean Daly Lynn, Katy Pedlow

**Affiliations:** ^1^ School of Health Sciences Ulster University Belfast UK

**Keywords:** AHP, allied health professional, experts by experience, higher education, service user involvement, SUI

## Abstract

**Background:**

The inclusion of Service User Involvement (SUI) in higher education, particularly within Allied Health Professions (AHP) programs, has been increasingly mandated by professional standards globally. This scoping review aimed to systematically map existing literature on active SUI in undergraduate AHP education to identify types of involvement activities, how involvement is measured and to identify gaps in the current evidence base.

**Methods:**

Using PRISMA‐ScR, a comprehensive search was conducted across five major databases; OVID Medline, OVID Embase, CINAHL, Scopus, and PsycINFO alongside grey literature. Analysis of results was framed using Towle's taxonomy and the Kirkpatrick evaluation model.

**Results:**

Twenty‐five studies met the inclusion criteria. Most involvement was with occupational therapy students (*n* = 13) followed by physiotherapy students (*n* = 7). Most studies documented SUI primarily in teaching delivery (*n* = 20), with less involvement in curriculum design (*n* = 2). The review highlighted limited engagement at higher levels of Towle's taxonomy, where they act as equal partners in educational processes. Regarding the Kirkpatrick model, most measured impact by immediate reactions (Level 1) or learning outcomes (Level 2), and few explored changes in student behaviour (Level 3). None of the included studies measured long‐term clinical outcomes (Level 4). Variability in terminology and practices was reported.

**Conclusion:**

This scoping review highlights the increasing value of (SUI) in undergraduate AHP education. Addressing barriers and standardising implementation are key to advancing meaningful engagement. Future research should explore the long‐term impact of SUI on clinical practice and patient outcomes.

**Patient and Public Involvement:**

A special interest group of service‐users were regularly consulted. They provided feedback on the search terms and all draft versions of the review.

## 1 Background

Service user involvement (SUI) in higher education has been driven by policy reform globally [1]. It is a government and professional body requirement that people who have lived experience of healthcare services are involved in higher education [2, 3, 4, 5]. Akin to patient and public involvement in which service users should be at the heart of service provision, design and delivery [6, 7, 8, 9], SUI supports the development of person‐centred healthcare professionals of the future [10, 11, 12, 13].

Terminology for SUI differs globally [14, 15, 16]. Different terms are used within healthcare to describe the people that they care for, confounding the terminology [15]. SUI variations include ‘expert patient’ [17, 18] or ‘experts by experience’ [19]. In the absence of an agreed term, this review will use SUI to include the people who have experience of using healthcare services as well as their caregivers. The hope is that this is an empowering term which provides a person‐centred lens that recognises the unique experience of each person as they interact with health services [1, 17].

Whilst service users are involved in education of healthcare professionals in placement activity, this is a more passive role [13] whilst ‘active’ SUI is viewed as service users fulfilling roles traditionally seen as academic. This could be curriculum design [20], delivery of teaching [21], assessment of students [22], or student recruitment [23]. This spectrum of activity is clarified by Towle's taxonomy demonstrating the different ways in which service users can be involved in healthcare education [14]. The taxonomy spans from level 1 where there is no direct involvement of the person, for example a written case study is shared with learners, up to level 6 where the service user is an equal partner to academic leads in the development of the curriculum at an institutional level (Table 1).

**Table 1:** Summary of Towle's taxonomy [14].

Level	Service user involvement activity	Passive or active
1	Video or transcript of patient experience is shared	Passive
2	Scripted patient encounter in a clinical setting	Passive
3	Patient shares their experience within a faculty‐directed teaching session	Active
4	Patients are teaching or evaluating students independently	Active
5	Patients are involved in many aspects of curriculum design and delivery	Active
6	Patients are involved at the institutional level and involved in decision‐making bodies	Active

In the current landscape, where global healthcare providers face unprecedented pressures, it is more crucial than ever for students to develop adaptability and engage with educational models that can swiftly respond to change and foster independent practice [24, 25]. Furthermore, provision of authentic learning experiences in a safe environment (University) can support the assimilation and retention of knowledge [26, 27, 28, 29]. How to best measure the impact of SUI is unknown, however the Kirkpatrick model [30, 31] provides an opportunity to explore it. Designed to evaluate the impact of training and education the model considers impact across 4 levels including student satisfaction (level 1), knowledge retainment (level 2), skill attainment (level 3) and outcomes (level 4). This model provides an opportunity for educators to explore not only if the students enjoyed the SUI experience, but more importantly whether it resulted in gained knowledge and skills and led to improved educational outcomes.

To date most evidence within the area of SUI in education has focused on medical education, social work, and nursing [32, 33, 34, 35], with lack of focus on allied health professional education [11, 12]. This work also highlighted the lack of long‐term measurement [32, 34] whilst identifying that service‐users involved in education may not be typical examples of the patients encountered in clinical practice, they do bring a valuable and unique perspective [35].

For the context of this review, Allied Health Professions are defined based on the Health and Care Professions Council (Table 2).

**Table 2:** HCPC.

HCPC list of 15 AHP roles and their relevant protected titles	
Radiographer Diagnostic radiographer Therapeutic radiographer	1
Paramedic	2
Chiropodist Podiatrist	3
Physiotherapist Physical therapist	4
Occupational therapist	5
Orthoptist	6
Prosthetist Orthotist	7
Art psychotherapist Art therapist Drama therapist Music therapist	8
Dietitian Dietician	9
Operating department practitioner	10
Speech and language therapist Speech therapist	11
Hearing aid dispensers	12
Clinical scientist	13
Biomedical scientist	14
Practitioner psychologist	15

### 1.1 Rationale

Given the increasing emphasis on person‐centred care and the diverse roles that Allied Health Professions (AHPs) play in healthcare delivery, understanding how service users contribute to the education is crucial. This scoping review aimed to systematically map the existing literature on SUI in undergraduate AHP education, identifying the range of involvement activities, how the impact of the involvement is measured, and any gaps that may exist in the current evidence base. This will help guide future research to better understand SUI in AHP education. This review set out to

1. Categorise the characteristics of SUI in undergraduate AHP education using Towles’ taxonomy.
2. Analyse how SUI is measured in undergraduate AHP education using the Kirkpatrick model.
3. Identify research needs for SUI in undergraduate AHP education.

### 1.2 Search Strategy

This review was guided by the PRISMA‐ScR (Preferred Reporting Items for Systematic reviews and Meta‐Analyses extension for Scoping Reviews) guidelines [36]. A literature search was conducted across five major databases: OVID Medline, OVID Embase, CINAHL, Scopus, and PsycINFO. Reference lists of identified papers were reviewed to identify further relevant papers.

The search strategy was developed to encompass the range of terms associated with service user involvement and their application within AHP education [12, 13]. The search terms were sent to a patient panel for approval (Supporting Information 1).

### 1.3 Eligibility Criteria

Inclusion criteria encompassed all study types with primary data that focused on active SUI within undergraduate AHP programs. Studies that involved simulated learning or actors, or those that primarily focused on clinical placements without an academic component [14], were excluded to maintain a clear focus on active service user engagement in educational settings. Service‐learning studies were viewed as placement learning, where the service users were receiving care or treatment from the students and were not actively involved in education of the students in a traditionally academic setting. Studies that included AHP students along with other healthcare professionals were included if the AHP students made up more than 50% of the total number of students in the group or if the AHP data had been separated.

Only publications written in English were included with no publication date restrictions applied. The aim of the broad inclusion criteria is to build an overall view of the landscape of literature relating to SUI in AHP education to guide future research needs. This is in keeping with Scoping Review guidance from Peters et al. [36].

The initial searches were conducted in June 2024 and repeated in October 2025. Any studies published after this date have not been included. No additional studies were identified from reference list screening.

### 1.4 Selection of Evidence Sources

Studies were imported into Covidence by CS.

**Figure 1:**
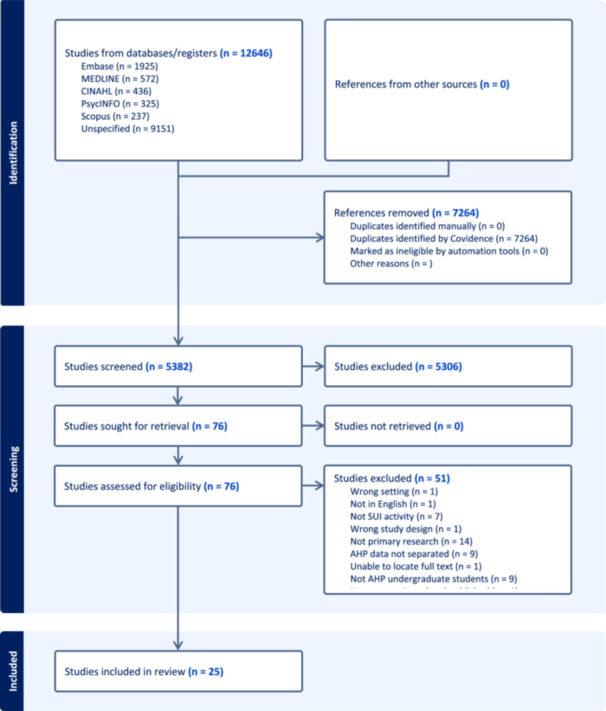
PRISMA flow diagram [37].

Title and abstract screening were duel screened (CS + JDL/KP). Agreement had to be reached by two researchers during this process with any disagreements checked by the third researcher to reach consensus. Figure 1 demonstrates the flow diagram as recommended in the PRISMA 2020 statement [37]. Full text screening was completed by the main author (C.S.) with a second author checking 20% (J.D.L./K.P.).

### 1.5 Data Charting and Synthesis

Data charting was completed in Covidence before being exported to Excel. Data extraction was completed by the primary author (C.S.) and included author details, year of publication, title of study, country of origin, aim of the study, data collection methods, participants, and SUI terminology; 20% of this data extraction was checked for accuracy by JDL. Once extracted, the primary author (C.S.) rated all studies using Towle's taxonomy of service user involvement [14] and against a modified Kirkpatrick four‐level training evaluation model to explore the impact of the interventions [31]. This rating was checked by J.D.L. and K.P.; any disagreements were resolved through discussion. Once all data were extracted, synthesis across the studies was completed to consider barriers and facilitators and service user feedback. A narrative synthesis approach was used to identify patterns and themes across the studies [38].

### 1.6 Main Results

Twenty‐five papers met the criteria [18, 21, 26, 39, 40, 41, 42, 43, 44, 45, 46, 47, 48, 49, 50, 51, 52, 53, 54, 55, 56, 57, 58, 59, 60].

Study characteristics including author details, year of publication, title of study, country of origin, aim of the study, data collection methods, participants, and SUI terminology were extracted. Studies were completed between 2011 year and 2025 year with 48% (*n* = 12) conducted in the United Kingdom, 24% (*n* = 6) conducted in Australia, 8% conducted in Canada (*n* = 2), 20% (*n* = 5) in other countries.

Most studies incorporated qualitative methodology (*n* = 23) [18, 21, 26, 40, 41, 42, 43, 44, 45, 46, 47, 48, 49, 50, 51, 52, 54, 55, 56, 57, 58, 59, 60], with some using supplementary quantitative data (*n* = 31) [43, 46, 54]. One study used quantitative data only utilising a comparative groups design [52]. Semi‐structured interviews, focus groups and surveys or questionnaires for feedback were the most common methods of data collection.

### 1.7 Participants

From the 25 included studies, 12 reported on data from the students only [26, 41, 43, 45, 46, 47, 48, 49, 51, 54, 55, 57], 6 from service users only [18, 21, 39, 50, 59, 60], and 2 collected data from academic staff only (Table 3) [53, 56].

**Table 3:** Participants of SUI in AHP education research.

Participant groups	Number (%)	References
AHP students only	12 (48%)	[26, 41, 42, 45, 46, 47, 48, 51, 54, 55, 57]
Service users only	6 (26%)	[18, 21, 39, 50, 59, 60]
Academic staff only	2 (9%)	[53, 56]
Service users and AHP students	2 (9%)	[40, 44]
AHP students and academic staff	2 (9%)	[43, 49]
Service users, staff and AHP students	1 (4%)	[52]

The most common academic programme was Occupational therapy (*n* = 13) [26, 39, 43, 44, 45, 46, 47, 50, 53, 54, 55, 56, 57, 58, 59]. Only one study [42] explored data relating to all AHP roles (Table 4).

**Table 4:** Data collected in relation to specific AHP roles.

AHP role	Number of studies	References
Occupational therapist	13 (57%)	[26, 39, 43, 44, 45, 46, 47, 51, 53, 54, 55, 56, 60]
Physiotherapist	7 (30%)	[26, 43, 45, 47, 55, 57, 58]
Therapeutic radiographer	5 (22%)	[18, 21, 43, 48, 50]
Speech and language therapist	3(13%)	[43, 44, 46]
Diagnostic radiographer	2 (9%)	[43, 51]
Drama therapist	2 (9%)	[40, 43]
Dietitian	2 (9%)	[43, 46]
Practitioner psychologist	2 (9%)	[43, 49]
Art therapist	1 (4%)	[43]
Biomedical scientist	1 (4%)	[43]
Clinical scientist	1 (4%)	[43]
Hearing aid dispenser	1 (4%)	[43]
Music therapist	1 (4%)	[43]
Operating department practitioner	1 (4%)	[43]
Paramedic	1 (4%)	[43]
Podiatrist	1 (4%)	[43]
Prosthetist/orthotist	1 (4%)	[43]

#### 1.7.1 SUI Terminology

**Figure 2:**
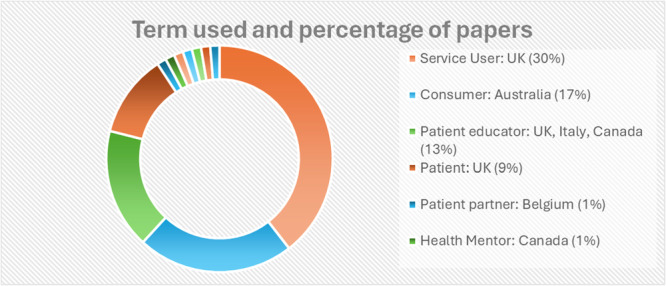
Pie chart of the terminology for the people involved in teaching, their location and percentage of papers.

The terminology used to describe people with healthcare experiences in AHP education varied across the studies (Figure 2). In Australian research, the term ‘consumer’ was most often reported [40, 51, 53, 56] whilst in the United Kingdom it was the term ‘service‐user’ [21, 43, 44, 49, 50, 52, 59].

#### 1.7.2 Rating Against Kirkpatrick

Impact, as measured by the Kirkpatrick model (Table 5), was mainly measured at the lower levels. Not all studies collected data applicable to the Kirkpatrick model, and some collected data at more than one level. Student, staff and service user satisfaction with the experience was measured by many of the studies (with some including data from more than one of these groups).

**Table 5:** Mapping against Towles taxonomy and adapted Kirkpatrick model.

Towles taxonomy—level of involvement	Number of studies
Levels 1 and 2 were considered ‘passive’ involvement and excluded from the review.	–
Level 3: Patient shares his or her experience with students within a faculty‐directed curriculum	12 (48%)
Level 4: Patient teacher(s) are involved in teaching or evaluating students	11 (48%)
Level 5: Patient teacher(s) are equal partners in student education, evaluation and curriculum development	2 (9%)
Level 6: Patients are involved at the institutional level	1 (4%)
Not applicable to the study	3 (13%)
**The adapted Kirkpatrick model**	
Level 1a: Students view of the experience such as satisfaction	5 (22%)
Level 1b: Service user perspectives	8 (35%)
Level 1c: Staff views on SUI	5 (22%)
Level 2: The SUI involvement resulted in an increase in skills, knowledge or attitudes	9 (35%)
Level 3: SUI resulted in an improvement in student behaviour	1 (4%)
Level 4: A measured change in the health and well‐being of the people who receive care from the students involved	0 (0%)

### 1.8 Rating Against Towles

A wide range of approaches to SUI were identified. The most common approach was SUI in teaching delivery (*n* = 20), with a smaller subset of studies utilising SUI in assessment (*n* = 4) [51, 52, 57, 59] and curriculum design (*n* = 2) [41, 50]. The involvement of service users in the recruitment and selection of students was not found. Some studies were surveys across regions and therefore the focus and results spanned across multiple levels of the taxonomy rather than one specific level of involvement.

According to Towle's taxonomy [14], most SUI activities were classified at the lower levels (*n* = 18) [18, 21, 26, 41, 43, 44, 45, 46, 47, 48, 49, 50, 53, 54, 55, 56, 57, 58, 59, 60] primarily involving service users sharing their experiences in the classroom via stories or lectures (*n* = 10) (3). Some studies included involvement across more than one level (*n* = 4) [49, 54, 58, 59].

### 1.9 Barriers and Facilitators to SUI in AHP Education

Remuneration for service‐users was frequently identified as a barrier [43, 52, 54], with a range of renumeration approaches reported. For example, some were paid as a casual lecturer under a version of a part‐time contract [54], whilst others were involved voluntarily, and some were given token gifts such as gift cards. The authors often expressed difficulty in this area and most expressed a wish to properly remunerate service users [43, 52, 54]. Chambers and Hickey [43] specifically mentioned caution regarding the impact that payments might have on other sources of income for the service users. Others raised concerns relating to the emotional health of service users as a result of their involvement in education [19, 42, 48, 56]. The positive experiences were also identified including the positive emotional impact of the educational experience [48], with recounting their experiences cathartic and rewarding. Some reported the positive impact of ‘giving back’ to the healthcare community [19, 43, 48, 56]. Personal development was identified as a valuable benefit to service users, such overcoming fear of public speaking [48, 56].

## 2 Discussion

This scoping review aimed to systematically map the existing literature on SUI in undergraduate AHP education identifying. The review identified that most SUI within AHP education is occurring in teaching and delivery within the lower levels of Towle's taxonomy.

Terminology used to describe the concept varied across regions, reflecting differences in policy frameworks and cultural interpretations. Despite this variation, adopting a single, consistent term would improve clarity, support comparative analysis, and strengthen communication among researchers, practitioners, and policymakers. Among the terms identified, Service User Involvement emerged as the most commonly used and widely understood. Therefore, it could be proposed as the preferred terminology going forward, ensuring a shared language that facilitates collaboration, enhances policy coherence, and reinforces the recognition of individuals’ active roles in shaping services that affect them.

Although the volume of research has not significantly increased in the past decade, the focus of studies has notably shifted from addressing initial concerns about incorporating service users in education to exploring best practices for meaningful involvement. Earlier studies often centred on the logistics of SUI [43] whilst more recent work emphasises coproduction, quality engagement and long‐term impact.

The impact on service users has also remained mixed across the studies. Previous research focusing on SUI in health education identified the positive impact on both empathy and professional identity. However, findings specific to AHP students are mixed. For instance, Ferri et al. [45] found significant pre/post changes in empathy and professional identity among medical and nursing students following SUI interventions but not among AHP students suggesting a positive ceiling effect for already high baseline scores. Similar findings in other studies [44] suggest the need for more targeted research within AHP education to fully understand these dynamics.

The impact of service user involvement was mixed, highlighting the need for careful consideration moving forward. The barriers and facilitators identified within this review suggest that the impact of service user involvement is far from straightforward. A tension between the desire to provide fair compensation for service user involvement and the potential unintended consequences that payment may introduce, particularly regarding its impact on other income sources is noted. Emotional wellbeing also emerges as a key consideration, as participation can evoke difficult feelings for some individuals. However, the evidence also points to meaningful benefits: many service users described involvement as emotionally affirming, valuing the chance to share their experiences, contribute to healthcare improvement, and pursue personal growth. Taken together, these insights suggest that while service user involvement offers significant potential, its success depends on careful planning and robust support that address both financial and emotional dimensions to enhance benefits and mitigate risks.

This review underscores the value of SUI at lower levels of Towles taxonomy involving storytelling, answering questions and assessing clinical or academic skills. These methods have demonstrated benefits in student learning particularly around professionalism and communication [26, 40, 42, 43, 44, 45, 46, 47, 48, 49, 52, 55, 58, 60]. Whilst all involvement is meaningful, it would be encouraged to ensure SUI opportunities are provided across all levels of the taxonomy ensuring SUI is fully integrated as partners in the educational process. This limited engagement may restrict the potential benefits of SUI, as higher levels of involvement, such as partnership and co‐production, are likely to yield more significant impacts on both student learning and patient care. Measuring impact of involvement remains a challenge. Most studies rely on Kirpatrick Level 2 outcomes (knowledge, skills, attitudes) [26, 44, 45, 47, 54, 58], whilst only one study [54] explored the impact of SUI on changing student behaviours (level 3). None assessed level 4 outcomes likely due to complexity and ethical considerations associated with restricting educational interventions. Nonetheless, robust longitudinal studies using validated frameworks are needed to explore long term outcomes of SUI in AHP education.

A commonly cited challenge was the lack of institutional guidance for integrating service users into educational settings. Enhanced reporting standards and clearer implementation frameworks would support more consistent practice and higher quality research. Future work should focus on evaluating the long‐term clinical impact of SUI and aim to develop evidence that can inform Level 4 Kirkpatrick outcomes.

A potential limitation of this review was the exclusion of literature focusing on the lower levels of passive service user involvement. While this decision ensured a focused analysis of active SUI, it may have excluded relevant insights. Nonetheless, the authors contend that clearer boundaries are needed to distinguish active involvement from passive exposure, supporting the development of more rigorous definitions and models of SUI in education. Furthermore, the inclusion of papers only in the English language is a limitation of this review. Whilst there was a range of countries represented, the majority of studies were completed within the United Kingdom.

## 3 Conclusion

This scoping review highlights the growing recognition and value of SUI in undergraduate AHP education. While the inclusion of service users in AHP education is increasingly recognised as beneficial, the findings suggest that much of the current practice remains at a basic level of involvement. Addressing barriers such as remuneration, developing institutional guidance, and adopting consistent reporting frameworks are essential next steps. Additionally, there is a need for studies that assess the long‐term impact of SUI on students’ clinical performance and its subsequent effect on patient care. By addressing these gaps, AHP education can evolve towards a more inclusive, patient‐centred approach that better prepares future professionals for the complexities of individualised healthcare practice.

## Author Contributions

**Chloe Shand:** conceptualisation (lead), methodology (lead), writing – original draft (lead), formal analysis (lead), review and editing (equal). **Jean Daly Lynn:** writing – original draft review and editing (supporting), formal analysis (supporting), supervision (equal). **Katy Pedlow:** writing – original draft review and editing (supporting), formal analysis (supporting), supervision (equal).

## Funding

The authors received no specific funding for this work.

## Ethics Statement

The authors have nothing to report.

## Conflicts of Interest

The authors declare no conflicts of interest.

## Supporting information

PRISMA 2020 checklist 1.

supmat.

Supplementary 2 data extraction table.

Supplementary 3 PRISMA ScR Fillable Checklist.

## Data Availability

Additional data will be available on request to the corresponding author.
