# Supplementary material for: A Scoping Review of Active Service User Involvement in Undergraduate Allied Health Professions Education
Source: Health Expect. 2026 Feb 4;29(1):e70575. doi: 10.1111/hex.70575 (PMC12873453; doi:10.1111/hex.70575)
Supplement: Supplementary file 2 — supmat. [file HEX-29-e70575-s002.docx]

Supplementary Document 1: Search Terms

| **#** | **Search**  **24/06/2024** | **OVID Medline Results** |
| --- | --- | --- |
| 1 | Patient Participation/ | 30084 |
| 2 | Community Participation/ | 48898 |
| 3 | Stakeholder Participation/ | 4498 |
| 4 | (“service user involvement” or “patient instructors” or “patient educators” or “patient teachers” or “patient-as-teacher” or “patient participation” or “community participation” or “patient involvement” or “patient engag*” or “community engag*” or “patient cooper*” or “community cooper*” or “patient collaborat*” or “community collaborat*” or “patient represent*” or “community represent*”).mp. | 73176 |
| 5 | 1 OR 2 OR 3 OR 4 | 76972 |
| 6 | Allied Health Personnel/ed | 14898 |
| 7 | Interprofessional Education/ | 586 |
| 8 | Allied Health Occupations/ed | 358 |
| 9 | Psychodrama/ed | 14 |
| 10 | Music Therapy/ed | 93 |
| 11 | Nutritianists/ed | 206 |
| 12 | Operating Room Technicians/ed | 159 |
| 13 | Osteopathic Physicians/ed | 69 |
| 14 | Osteopathic Medicine/ed | 1285 |
| 15 | Physical Therapy Modalities/ed | 1719 |
| 16 | Radiography/ed | 358 |
| 17 | Speech Therapy/ed | 257 |
| 18 | Podiatry/ed | 400 |
| 19 | Occupational Therapy/ed | 1655 |
| 20 | Technology, Radiologic/ed | 1225 |
| 21 | Radiotherapy/ed | 25 |
| 22 | (“AHP education” or “AHP curriculum” or “allied health education” or “allied health curriculum” or “allied health professional education”).mp. | 291 |
| 23 | ((education or curriculum or undergraduate or student or training or course or study) **adj3** ("art therap*" or "drama therap*" or "music therap*" or chiropod* or podiatr* or dietitian or "operating department practi*" or orthopt* or osteopath* or paramedic* or physiotherap* or prosthetist* or orthotist* or radiograph* or radiotherap* or "speech and language therap*" or "speech therap*" or "occupational therap*" or "hearing aid dispenser*" or "clinical scien*" or "biomedical scien*" or psychologist* or dietician*)).mp. | 23030 |
| 24 | 6 OR 7 OR 8 OR 9 OR 10 OR 11 OR 12 OR 13 OR 14 OR 15 OR 16 OR 17 OR 18 OR 19 OR 20 OR 21 OR 22 OR 23 | 43479 |
| 25 | 5 AND 24 | 286 |
