# Supplementary material for: A Scoping Review of Active Service User Involvement in Undergraduate Allied Health Professions Education
Source: Health Expect. 2026 Feb 4;29(1):e70575. doi: 10.1111/hex.70575 (PMC12873453; doi:10.1111/hex.70575)
Supplement: Supplementary file 3 — Supplementary 2 data extraction table. [file HEX-29-e70575-s003.docx]

| **Author** | **Title** | **Country** | **Aim of study** | **Study design** | **Participants who provided data (n=)** | **Terminology** | **AHP profession** |
| --- | --- | --- | --- | --- | --- | --- | --- |
| Arblaster 2018 | Learning from consumers: An eDelphi study of Australian mental health consumers' priorities for recovery-oriented curricula | Australia | Identify consumer priorities for curricula and ways they want to participate in entry-level student education | Qualitative: Survey/Questionnaire | Service user/patient teacher/experts  (n=14) | Consumer | Occupational Therapist |
| Arias  2020 | A Space to Speak: Therapeutic Theater to Address Gender-Based Violence | United States | Describe creation and performance process of theater piece and its impact on performers/audience | Qualitative: Focus group | Service user/patient teacher/experts; Undergraduate AHP students; Audience members (unknown n)  (n=8) | Students told their stories of gender-based violence | Drama Therapist |
| Balandin 2011 | The involvement of people with lifelong disability and communication impairment in lecturing to speech-language pathology students | Norway | Explore how final-year speech-language pathology students and new graduates view impact of lectures by people with lifelong disabilities | Qualitative: Focus group | Undergraduate AHP students; final-year students and new graduates  (n=14) | People with [named condition/ailment/disease] | Speech and Language Therapist |
| Bazin 2016 | Learning with expert patients – can this support the development of clinical skills? | UK | To explore physiotherapy students' experiences when developing assessment skills during learning opportunities with expert patients. | Qualitative: Survey/Questionnaire | Undergraduate AHP student/s (n=26) | Expert patient | Physiotherapist |
| Carmichael  2017 | Expert patient perspectives on radiotherapy: a phenomenological comparison | Australia | Contrast perspectives of ‘educator’ patient and ‘lay’ patient on teaching sessions | Qualitative: Interview | Service user/patient teacher/experts  (n=2) | Expert patient | Therapeutic Radiographer |
| Chambers 2012 | Service user involvement in the design and delivery of education and training programmes leading to registration with the Health Professions Council. | UK | To explore the current involvement of service users in pre-registration education and training programmes approved by the HPC. Objectives: understanding nature and extent of involvement, analyzing types of involvement activities, situating findings in literature. | Mixed method – 4 stages: 1) Literature review, 2) Matrix of benefits/barriers to SUI, 3) Online questionnaire and case studies, 4) Consensus workshop | Undergraduate AHP students; Academic staff; Mixture of above  191 survey responses; 21 academic staff focus groups; 19 student focus groups | Service user | All AHP groups |
| Cleminson  2013 | Service user involvement in occupational therapy education: an evolving involvement | UK | Summary of teaching sessions with service user involvement | Summary of teaching sessions | Service user/patient teacher/experts; Undergraduate AHP students; Mixture of above  1 service user; unknown number of students | Service user | Occupational Therapist |
| Etenaille 2023 | Evaluation of inter-professional seminar involving patient-partners and caregivers | Belgium | To assess contribution of interprofessional education (IPE) for pre-graduate students, with seminars co-facilitated by patient partners and caregivers to enhance interprofessional communication and patient involvement in decision-making | Qualitative: Survey/Questionnaire | Undergraduate AHP students  66 physiotherapy students; 29 occupational therapy students | Patient partner | Physiotherapist; Occupational Therapist |
| Ferri, 2023 | Evaluation of an interprofessional education intervention in partnership with patient educators | Italy | Evaluate whether an IPE intervention with patient educators increases readiness for interprofessional learning and empathy in health sciences students | Mixed methodology: Quantitative and Qualitative | Undergraduate AHP students  36 AHP students (310 total healthcare students) | Patient educator | Occupational Therapist; Dietitian; Speech and Language Therapist; Mixed AHP groups |
| Flood 2018 | Service user involvement in radiotherapy and oncology education; the patient perspective | UK | Determine service user perspectives on sharing personal cancer treatment experiences with students | Qualitative: Interview | Service user/patient teacher/experts  (n=7) | Service user | Therapeutic Radiographer |
| Henricksen 2011 | Learning from patients: students' perceptions of patient-instructors | Denmark | Explore added value of using patients as instructors and how it is constituted | Focus group | Undergraduate AHP students  (40 total: 23 physio, 17 OT) | Patient instructor | Physiotherapist; Occupational Therapist; Mixed AHP groups |
| Hodgson 2014 | Patient and carer involvement in the radiotherapy curriculum: The impact on students' professional development | UK | Explore how therapeutic radiography students learn from patient/carer experiences and contribute to professional development; secondary aim to develop curriculum further | Qualitative: Interview | Undergraduate AHP students  (n=18) | Patient | Therapeutic Radiographer |
| Holttum, 2010 | Perceived improvements in service user involvement in two clinical psychology courses | UK | Examine staff and trainee ratings of service user/carer involvement, course friendliness, and barriers/opportunities for involvement | Qualitative: Survey/Questionnaire | Undergraduate AHP students; Academic staff; Mixture of above  125 total: 44 trainee clinical psychologists; 81 staff | Service user | Practitioner Psychologist |
| Keenan  2014 | Service user involvement in cancer professionals’ education: perspectives of service users | UK | Explore motivations and experiences of service users’ involvement in radiotherapy/oncology education | Qualitative: Interview | Service user/patient teacher/experts  (n=5) | Service user | Therapeutic Radiographer |
| Logan 2018 | Mental health consumer participation in undergraduate occupational therapy student assessment: No negative impact | Australia | Investigate whether a mental health consumer as assessor impacts engagement, anxiety, and academic performance | Non-randomised experimental comparison | Undergraduate AHP students  (n=79) | Consumer | Occupational Therapist |
| Naylor  2015 | An exploration of service user involvement in the assessment of students | UK | Explore views of service users, academic staff, and students | Mixed: Focus group (academics/SUs) + email feedback from students | Service user/patient teacher/experts; Undergraduate AHP students; Academic staff; Mixture | Service user  4 in focus group (2 academics, 2 SUs); 5 student emails | Diagnostic Radiographer |
| Scanlon 2018 | Mental health consumer involvement in occupational therapy education in Australia and Aotearoa New Zealand | Australia | Map patterns of consumer involvement in OT programs across Australia and NZ | Qualitative: Survey/Questionnaire | Academic staff  (23 programs from 19 universities) | Consumer | Occupational Therapist |
| Scanlon 2022 | Learning from lived experience: Outcomes associated with students' involvement in co-designed and co-delivered recovery-oriented practice workshops | Australia | Evaluate outcomes for students involved in co-designed/co-delivered recovery-oriented workshops with Lived Experience Educators | Mixed methodology: RKI and CROP-Q; change over time with paired t-tests and qualitative student feedback | Undergraduate AHP students (90 students) | Individual with lived experience | Occupational Therapist |
| Ruitenberg  2015 | "How to do things with words" in health professions education | Canada | Explore what health professions students learn about professional language and communication in interprofessional groups with a patient, and how students reflect on their development of professional discourse | Journal reflections | Undergraduate AHP students  17 OT students; 6 Physiotherapy students (82 total healthcare students) | Health Mentors | Physiotherapist; Occupational Therapist |
| Solomon 2011 | Student perspectives on patient educators as facilitators of interprofessional education | Canada | Evaluate student perceptions of learning in patient-facilitated IPE event | Focus groups and questionnaires | Undergraduate AHP students  (27 focus groups, 138 questionnaires) | Patient educator | Physiotherapist; Occupational Therapist; Medicine; Nursing |
| Soon  2022 | Describing consumer involvement, recruitment, retention in Australian occupational therapy university education: A cross sectional survey | Australia | N/A | Qualitative: Survey/Questionnaire | Academic staff  (n=9) | Consumer | Occupational Therapist |
| Stephenson 2025 | Forensic Doctorate Trainee Views on an Expert by Experience (EbE)-Led Workshop: A Qualitative Exploration of Trainee Reflections | UK | To identify what trainees felt were the important outcomes of the workshop and gain insights on what was useful, what they had learnt, and impact on their work. | Non-randomised experimental study | Undergraduate AHP student/s (n=16) | Expert by experience | Practitioner Psychologist |
| Thomson  2011 | An evaluation of students' perceptions of a college-based programme involving patients, carers, and service users in physiotherapy education | UK | Evaluate student perceptions of programme outcomes and learning in order to improve/modify programme | Focus groups and interviews | Undergraduate AHP students  30 in focus groups; 7 in interviews (total 37) | Patient | Physiotherapist |
| Thomson  2013 | Service users’ perceptions regarding their involvement in a physiotherapy educational programme in the UK | UK | Explore service users' perspectives of involvement in college-based physiotherapy programme | Qualitative: Interview | Service user/patient teacher/experts | Patient educator  (n=8) | Physiotherapist |
| Walsh, 2016 | Learning from stories of mental distress in occupational therapy education | UK | Describe learning processes from personal experiences of mental distress when service users participate in occupational therapy education | Interviews and focus groups | Service user  9 total: 3 service-user educators; 3 tutors; 3 students | Service user | Occupational Therapist |
